# Prevalence and Genetic Diversity of Group A Rotavirus Genotypes in Moscow (2019–2020)

**DOI:** 10.3390/pathogens10060674

**Published:** 2021-05-30

**Authors:** Anton Yuzhakov, Ksenia Yuzhakova, Nadezhda Kulikova, Lidia Kisteneva, Stanislav Cherepushkin, Svetlana Smetanina, Marina Bazarova, Anton Syroeshkin, Tatiana Grebennikova

**Affiliations:** 1 Laboratory of Molecular Diagnostics, N. F. Gamaleya Federal Research Center for Epidemiology & Microbiology, 123098 Moscow, Russia; chekh-ks@mail.ru (K.Y.); nad007@rambler.ru (N.K.); lborisovna2007@yandex.ru (L.K.); cherepushkin1@gmail.com (S.C.); t_grebennikova@mail.ru (T.G.); 2 Laboratory of Biochemistry and Molecular Biology, Federal State Budget Scientific Institution “Federal Scientific Center VIEV”, 109428 Moscow, Russia; 3 Moscow Clinical Hospital No. 1 of Infectious Diseases, 125310 Moscow, Russia; ikb1@zdrav.mos.ru (S.S.); ikb_1@mail.ru (M.B.); 4 Department of Pharmaceutical and Toxicological Chemistry, Peoples’ Friendship University of Russia (RUDN University), 117198 Moscow, Russia; livmatter@mail.ru

**Keywords:** rotavirus A, genotype, acute gastroenteritis, DS-1-like, Wa-like

## Abstract

Group A rotavirus (RVA) infection is the leading cause of hospitalization of children under 5 years old, presenting with symptoms of acute gastroenteritis. The aim of our study was to explore the genetic diversity of RVA among patients admitted to Moscow Infectious Disease Clinical Hospital No. 1 with symptoms of acute gastroenteritis. A total of 653 samples were collected from May 2019 through March 2020. Out of them, 135 (20.67%) fecal samples were found to be positive for rotavirus antigen by ELISA. RT-PCR detected rotavirus RNA in 80 samples. Seven G-genotypes (G1, G2, G3, G4, G8, G9, and G12) and three P-genotypes (P[8], P[4], and P[6]) formed 9 different combinations. The most common combination was G9P[8]. However, for the first time in Moscow, the combination G3P[8] took second place. Moreover, all detected viruses of this combination belonged to Equine-like G3P[8] viruses that had never been detected in Russia before. The genotype G8P[8] and G9P[4] rotaviruses were also detected in Moscow for the first time. Among the studied rotaviruses, there were equal proportions of Wa and DS-1-like strains; previous studies showed that Wa-like strains accounted for the largest proportion of rotaviruses in Russia.

## 1. Introduction

Diarrheal diseases are one of the leading causes of morbidity and mortality among children under 5 years old all over the world, especially in developing countries with poor quality health care. Rotavirus infection is the most common cause of diarrheal diseases [1]. More than 200,000 children died from rotavirus in 2013 [2]. In 2019, a total of 780,497 cases of acute intestinal infections were recorded in the Russian Federation (compared to 816,012 cases in 2018), out of which only 37.1% were cases with an identified etiology; the group A rotavirus (RVA) was the most common causative agent. The economic damage from rotavirus infection amounted to more than USD 130 million in Russia in 2019 [3,4].

Following the introduction of rotavirus vaccines in many countries, there has been a decrease in all-cause diarrhea and rotavirus-related hospital admissions [1]. The criterion for successful vaccination is high coverage of the target population. The higher the vaccination coverage is, the more significant reductions in rotavirus-related hospital admissions will be [5]. Only one vaccine against rotavirus, RotaTeq, has been registered in the Russian Federation. To date, immunization against rotavirus infection does not cover all the regions of the Russian Federation, and has had no significant impact on the epidemiology of rotavirus countrywide. In some areas of Moscow, vaccination began in 2014, and was available in the entire city by 2018. Yet, vaccination coverage remains low; only 25–27% of newborns were vaccinated in 2018–2019 [3,4].

Rotavirus A is a non-enveloped virus belonging to the family Reoviridae, which contains a double-stranded RNA genome with 11 segments encoding six non-structural (NSP1-6) and six structural (VP1-4, VP6-VP7) proteins [6]. The binary classification of RVA is based on nucleotide sequence of the two genes which encoded structural proteins, glycoprotein (G) VP7, and protease-sensitive protein (P) VP4, located on the outer capsid. Currently, there are 36 G-genotypes and 51 P-genotypes recognized [7]. Worldwide, the most common genotype combinations of human rotaviruses are G1P[8], G2P[4], G3P[8], G4P[8], G9P[8], and G12P[8] [8]. In Russia, viruses related to five of these combinations prevailed, excluding G12P[8]. During 2009–2014 in Moscow, the G4P[8] was the predominant genotype, while G9P[8] was the most frequent genotype in 2018–2019 [9,10].

The Moscow agglomeration is the largest in Europe, as its population ranges from 17 to 25 million people [11]. The high population density, the constant influx of migrants from other regions of Russia and from the post-Soviet countries as well as the impact of global tourism create favorable conditions for reassortment of viral genes and changes in circulating rotavirus genotypes. Such changes were also observed in some countries, following the introduction of vaccination [12,13,14].

The aim of our study was to explore the diversity of rotavirus genotypes circulating in Moscow in 2019–2020 and to monitor possible changes as a result of vaccination.

## 2. Results

**Figure 1:**
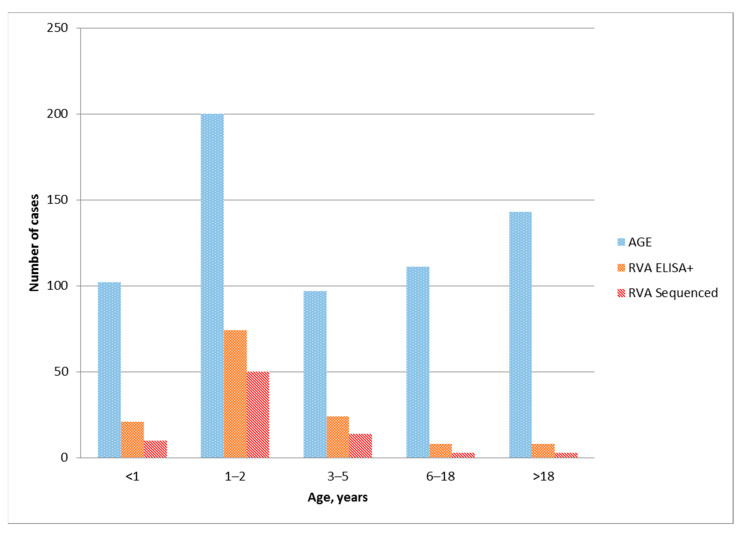
Distribution of acute gastroenteritis cases, rotavirus ELISA-positive samples and genotyped RVA among age groups.

From May 2019 to March 2020, a total of 653 samples were collected from patients with AGE symptoms. Out of them, 135 (20.67%) fecal samples were positive for rotavirus antigen by ELISA. RT-PCR detected rotavirus RNA in 80 samples. The age composition of patients with acute gastroenteritis, positive by ELISA, and presenting a specific genotype of group A rotavirus samples is shown in Figure 1.

Nucleotide sequences of the VP7 and VP4 genes were identified for 78 out of 80 samples; only the VP7 sequence was identified for 2 samples. The obtained sequences were deposited in the GenBank database under the numbers MT939912–MT940069 (Table S1).

**Figure 2:**
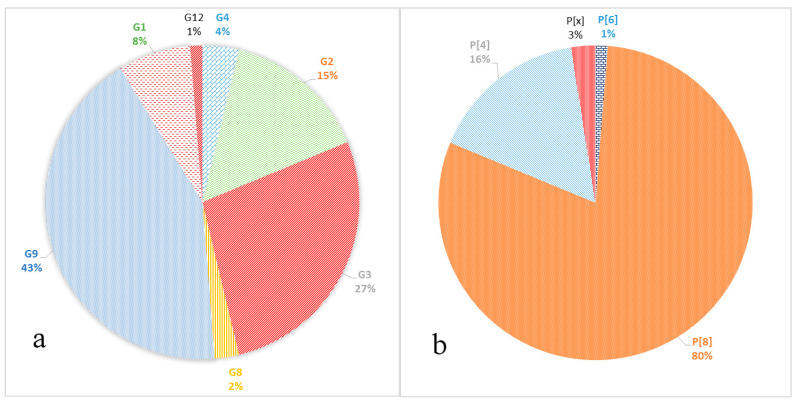
Frequency of VP7 (**a**) and VP4 (**b**) genotypes in the samples.

The following genotypes of RVA were identified. Seven G-genotypes G1, G2, G3, G4, G8, G9, and G12 were detected (Figure 2a). The genotypes G9 and G3 were detected in 43% and 27% of cases, respectively. Genotype G2 was detected in 15% of cases; genotype G1 in 8%. Minor genotypes G4, G8, and G12 were detected in 4%, 2%, and 1% of cases, respectively.

Three P-genotypes were detected: P[8], P[4], and P[6] (Figure 2b). The P[8] prevailed, accounting for 80% of all identified samples. Genotype P[4] was detected in 16% of cases; P[6] was detected in 1% of cases. In two cases (3%), the P genotype could not be identified P[x].

**Figure 3:**
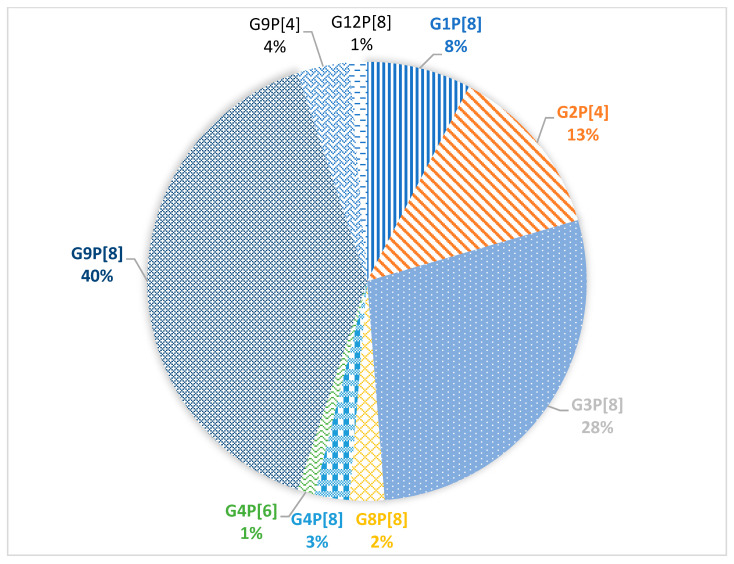
Prevalence of G/P genotype combinations of group A rotavirus.

Combinations of G/P genes were identified for 78 samples. In total, 9 different variants of gene combinations were detected (Figure 3).

The dominant combinations G9P[8] and G3P[8] were detected in 40% and 28% of cases, respectively. In 13% of cases, the G2P[4] combination was detected. The G1P[8] combination was detected in 8% of cases. Minor combinations G9P[4], G4P[8], G8P[8], G4P[6], and G12P[8] were detected in 4%, 3%, 2%, 1%, and 1% of cases, respectively.

**Figure 4:**
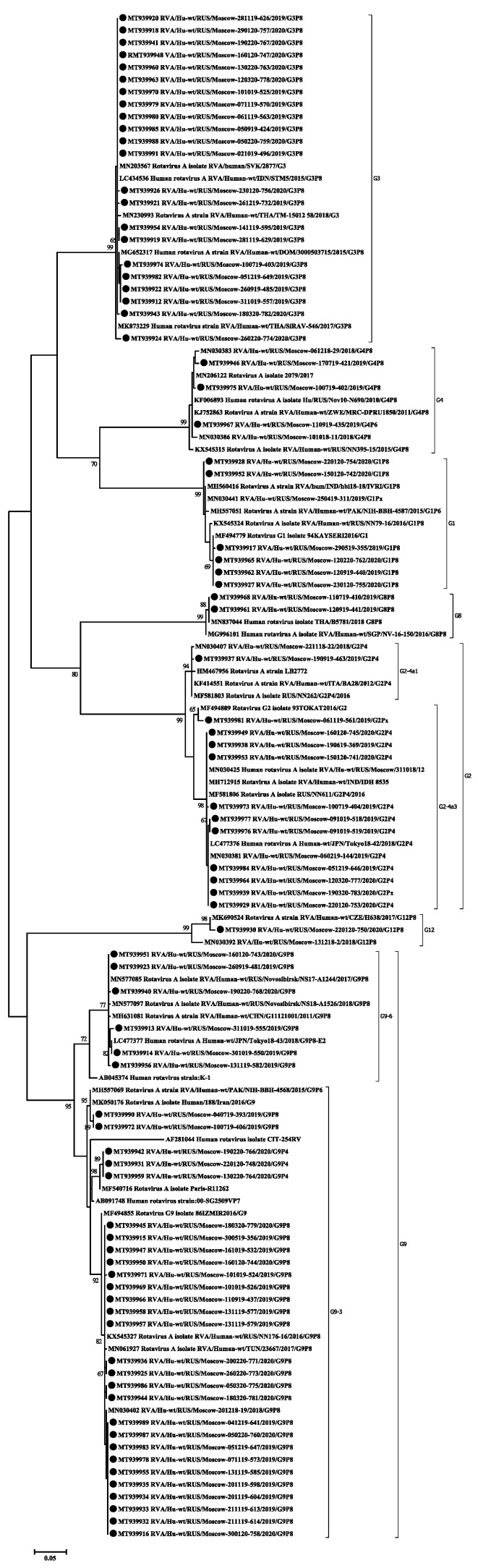
Phylogenetic tree of human rotavirus A strains based on partial VP7 nucleotide sequences. Bootstrap confidence limits are shown at each node; values less than 65 are not shown. Sequences from this study are marked by ●.

**Figure 5:**
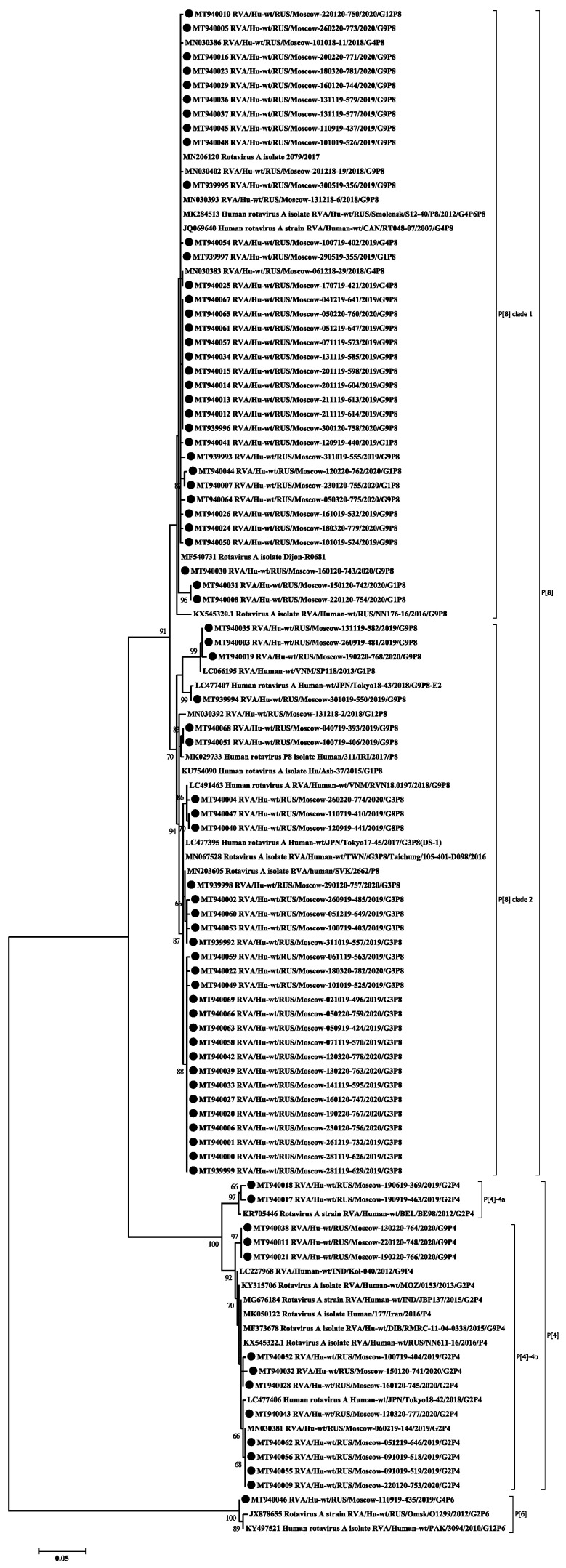
Phylogenetic tree of human rotavirus A strains based on partial VP4 nucleotide sequences. Bootstrap confidence limits are shown at each node; values less than 65 are not shown. Sequences from this study are marked by ●.

Based on the obtained partial nucleotide sequences of the VP7 and VP4 genes as well as on the sequences of these genes from the GenBank database, multiple alignments were made and phylogenetic dendrograms were plotted separately for each gene (Figure 4 for VP7 and Figure 5 for VP4).

VP7. The G1 genotype included 6 strains with 98–100% nucleotide identity. They belonged to the G1-2 lineage of G1-genotype. Twelve strains of the G2 genotype are divided into two unequal clades. One strain belonged to the G2-4a1 lineage, eleven strains with nucleotide identity level reaching about 96–100% belonged to the G2-4a3 lineage. The G3 genotype included 22 studied strains with 98–100% nucleotide identity. Three strains belonged to the G4 genotype with 99% nucleotide identity. The two detected strains of genotype G8 had more than 99% nucleotide identity. Most (28 out of 34) strains involving the G9 genotype belonged to lineage 3 of this genotype. The nucleotide identity in this group was 96–100%. The smaller clade contained strains with 99–100% nucleotide identity belonging to lineage 6 of genotype G9. The only strain of the genotype G12 formed a separate group.

VP4. All 64 strains of P[8] genotype belonged to the P[8]-3 lineage. Within the clade P[8], two practically equivalent subclades can be distinguished. A slightly larger part, 34 strains out of 64, had 97–100% nucleotide identity. The second subclade contained 30 strains out of 64. The level of nucleotide identity in this group was 96–100%. Thirteen strains belonging to genotype P[4] were distributed between two subclades. Two strains belonged to the P[4]-4a lineage and 11 strains to the P[4]-4b lineage. A single strain of genotype P[6] formed a separate group.

## 3. Discussion

The largest number of ELISA-positive rotavirus samples were in the group of patients from one to three years old, while in the group of children under 1 years old in several times lower, 74 and 21 cases were found, respectively (Figure 1). This is consistent with previous evidence showing that children hospitalized for rotavirus are older in countries with low child mortality rates than in countries with high rates [15]. All 135 antigen-positive samples were sent to the molecular diagnostics laboratory for genotyping. Group A rotavirus was genotyped in 80 samples. Genotyping of only 80 out of 135 samples positive in ELISA can be associated with both the low amount of viral RNA in the samples under study and the low sensitivity of the RT-PCR used. As expected, the largest number of samples with an identified rotavirus genotype was obtained from children aged 1 to 3 years. In total, 74 (92.5%) genotyped viruses were obtained from samples from children under 6 years old (Table 1).

In addition, 3 genotyped viruses were obtained from samples in groups of patients aged 6–18 and over 18 years. The viruses detected in these age groups were of two types G: G2 and G9, and P: P[4] and P[8] (the P-type was not identified for one of the samples).

Although rotavirus affected mainly children under 5 years of age, adult cases are also known. Three outbreaks of rotavirus infection among adults were reported in the United States in 1998–2000. In these outbreaks, the detected viruses belonged to the G2 genotype; the same genotype was detected in adult patients in Japan [16]. The outbreak among adults in the United States in 2013 was caused by a virus with the genotype G12P[8] [17]. In the study conducted in Sweden, the genotype G9[8] was found in most adult patients, similar to G1P[8] (most common in children at the same period) and G2P[4] [18]. The detection of certain genotypes of rotaviruses in adult patients, which often do not coincide with the dominant genotypes in children, though detected during the same period and in the same area, can be explained by virulent properties of these viruses. [16]. If this is the case, then in the future we will see an increase in cases of rotavirus infection in adults associated with the replacement of Wa-like viruses with DS-1-like ones.

The VP7 gene (G-type) was sequenced in all the 80 studied rotaviruses. Seven different genotypes were detected: G1, G2, G3, G4, G8, G9, and G12 (Figure 2a). The dominant genotype G9 was detected in 43% of cases. The G9 genotype has been detected in Russia since 2002. Since then, its proportion among other G-types has been increasing. From 2014 to 2016, in Nizhny Novgorod (European part of Russia) it was the main genotype, accounting for 35.4–45.9% of cases [19]. In Moscow, until 2012–2013, when it was unexpectedly detected in 30% of cases, it had been relatively rare [10]. However, in 2018–2019, this genotype was already dominant and accounted for 35.8% of cases [9]. In our study, most of the G9 genotype strains belong to lineage 3 of this genotype, according to the classification proposed by Phan [20]. This lineage included the studied strains with combinations of genotypes G9P[8] and G9P[4]. The majority of G9 strains currently known worldwide belong to this lineage. The strains in our study turned out to be similar to strains from Russia, Pakistan, Iran, France, Turkey, and Tunisia (Figure 4). The possible transmission of viruses of this genotype into the central part of Russia from Turkey was previously discussed [18]. Six G9 strains obtained in this study belonged to the lineage 6 of this genotype, all strains with the G9P[8] combination. Initially, this lineage included strains isolated from humans and pigs from China and Japan [20,21]. In Russia, strains of this lineage were previously detected in Siberia. Taking into account the geographic proximity of China as well as a large flow of tourists from China to Moscow, we can make respective assumptions regarding the origin of this lineage in Russia.

The second most frequent genotype G3 was detected in 27% of cases. In 2009–2014 in Moscow, this genotype was detected in fewer than 5% of cases [10]. The G3 sequences obtained in this study are closely related to the strains from Thailand, Slovakia, the Dominican Republic, and Indonesia. Viruses from the Dominican Republic and Indonesia are Equine-Like G3 strains; such strains have been found in humans since 2013 and were supposedly transmitted by horses [22,23].

The frequency of the G2 genotype remained practically unchanged compared to the previous season, 15% and 16.4%, respectively [9]. Earlier in Moscow, the proportion of this genotype fluctuated around 3% [10]. Most of the studied strains, together with strains from Russia, India, Japan, and Turkey, belonged to the G2-4a3 lineage that has spread worldwide since 2000 [24,25]. One strain belonged to the G2-4a1 lineage, together with strains from Russia, Italy, and the United States. This lineage had dominated in the world until the mid-2000s [26].

The proportion of the G1 genotype in Moscow has remained approximately constant since 2009, accounting for about 10%. The studied strains form a group with strains previously isolated in Russia, as well as strains from India, Pakistan and Turkey. All studied strains belong to the 1-2 lineage, like the vaccine strain in the Rotarix vaccine. Earlier in Russia, strains related to the 1-1 lineage were also detected [27].

The frequency of G4 genotype has decreased against last year by 7 times, from 4% to 27%, respectively [9]. Earlier in Moscow, G4 was the dominant genotype and accounted for about 40% of cases [10]. Also, this genotype had been dominant in other regions of Russia until 2015–2016 [4,19,28].

Minor genotypes G8 and G12 were detected in 2% and 1% of cases, respectively. The G8 genotype has not been previously detected in Moscow. Viruses with genotype G8 are common in cattle and also cause disease in humans in Africa and Asia [29]. The studied viruses of the G8 genotype were similar to strains from Thailand and Singapore. Viruses belonging to the G12 genotype are rare in Russia [28].

The VP4 gene (P-type) was sequenced for 78 studied rotaviruses; in two cases the P-type remained unknown. The main genotypes and their percentage have remained virtually unchanged since last season [9]. The most common P-genotype was the P[8] genotype in 64 out of 78 cases. Four lineages were described for the P[8] genotype previously; all samples studied by us belonged to lineage P[8]-3 [30]. Earlier in Russia, replacement of the circulating lineage 1 with lineage 3 was reported [31]. The phylogenetic analysis of the studied sequences showed that they were divided into two clades, the bootstrap value was 91. The first clade included viruses with the following G-types: G1, G4, G9, and G12. This clade also included viruses previously isolated in the European part of Russia and strains from France and Canada. The second clade included all the studied viruses with a combination of G3 and G8, and only six strains with the G9 genotype. This clade also included mainly Asian viruses, except for the strain from Slovakia.

The P4 genotype was detected in 13 cases. Two of them belonged to the P[4]—4a sublineages and 11 to the P[4]—4b sublineages. The increase in the 4b sublineage in the 2010s was recorded around the world [26]. The P6 genotype was detected in one case and was closely related to the strains from Siberia and Pakistan. Viruses of this genotype are common in sub-Saharan Africa, but are occasionally detected in other regions of the world [32].

**Figure 6:**
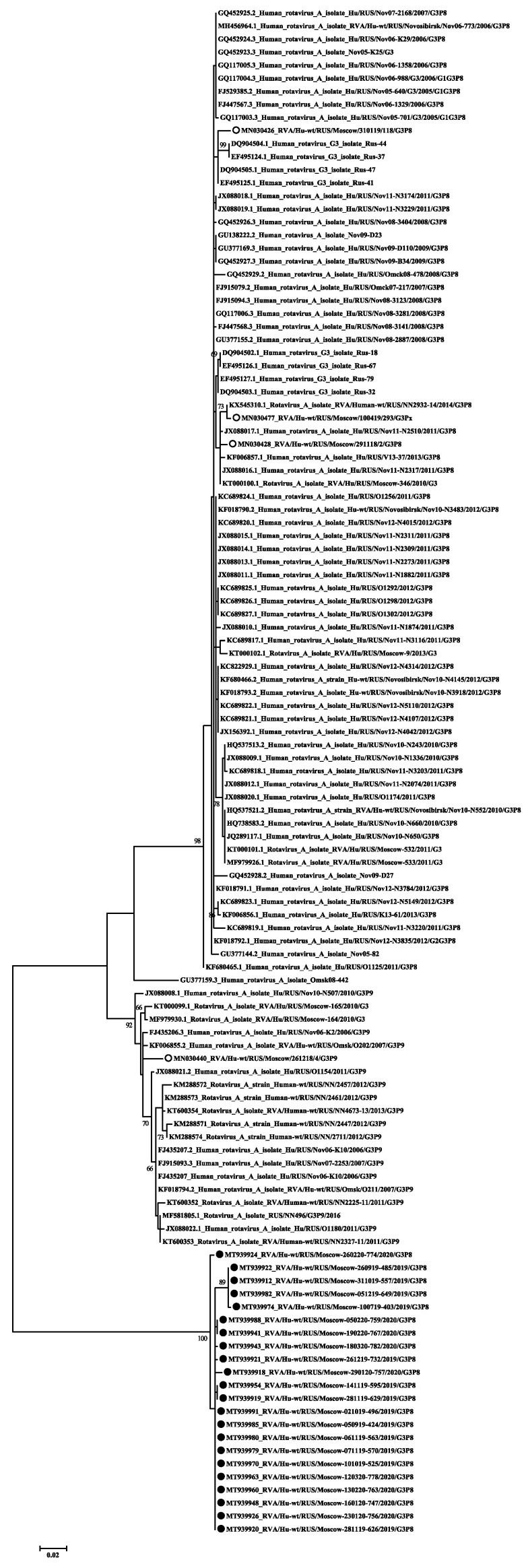
Phylogenetic tree of Russian genotype G3 human rotavirus A strains based on partial VP7 nucleotide sequences. Bootstrap confidence limits are shown at each node; values less than 65 are not shown. Sequences from this study are marked by ●, sequences from Moscow 2018–2019 are marked ○.

Compared to the 2018–2019 season, the dominant G/P genotype combination did not change, it is G9P[8] accounting for 40% of all cases [8]. However, the second prevalent combination was G3P[8] (28% of all cases); last year it was detected only in 4.9% of cases. Moreover, all the studied G3P8 viruses turned out to be closely related to the Equine-like G3 strains widespread in Southeast Asia and having a DS-1-like genotype [22,23]. Earlier in Russia (and in Moscow, in particular) only typical human Wa-like viruses with a combination of G3P[8] were detected (Figure 6).

Accordingly, the second combination of last season G4P[8] (29.3%) was detected in only 3% of cases during this season. Previously, viruses with this combination were the most common in Moscow [10].

The proportion of G2P[4] and G1P[8] combinations remained practically unchanged over the past season [8]. The virus with the G12P[8] combination was detected in a single case last season; this year, it was also detected only once. Viruses with combinations of the G4P[6], G8P[8], and G9P[4] genotypes were also detected, though they had never been detected in Moscow before. Viruses with the G4P[6] combination are found both in humans and pigs; they belong to the Wa-like genotype [33]. In Russia, this genotype combination was detected previously in the Siberian region [28]. Viruses with the G8P[8] genotype combination have been detected in people in Africa and Asia, where they cause disease not only in children, but also in adults [29,34]. Viruses with the G9P[4] combination are widespread in Asia and South America, though sometimes they are detected in Europe [35]. In Russia, viruses with combinations of G8P[8] and G9P[4] were detected by PCR in Nizhny Novgorod, unfortunately they were not sequenced [25]. Viruses with G8P[8] and G9P[4] combinations belonged to DS-1-like strains [34,35].

We saw that viruses related to Wa-like, which accounted for 83% of all cases during the previous season, have been detected only in 50% of cases this year. The increase in the number of circulating DS-1-like viruses has been reported in many countries, possibly due to the introduction of vaccination [23,36,37]. It is not known whether the increase in DS-1-like viruses in Moscow is related to vaccination against rotavirus, as RotaTeq vaccine coverage remains below 30%. Alternatively, it may be associated with an increase in the DS-1-like viruses in the countries frequently visited by Moscow citizens, and then new variants of rotaviruses are brought to Russia. In general, it is necessary to monitor circulating strains, given the high rate of spread of previously rare viruses [21].

## 4. Materials and Methods

### 4.1. Samples

Fecal samples were collected from 653 patients aged from 1 month to 94 years; all of them presented symptoms of acute gastroenteritis (AGE) when admitted to Moscow Clinical Hospital No. 1 of Infectious Diseases. Samples were collected from May 2019 to March 2020. The samples were tested for the presence of rotavirus antigen by ELISA in the hospital laboratory, with a Rotavirus-antigen-ELISA-BEST kit (Vector-best, Novosibirsk, Russia). Rotavirus antigen-positive samples were transferred on the same day to the laboratory of molecular diagnostic of the The National Research Center for Epidemiology and Microbiology named after Honorary Academician N. F. Gamaleya of the Ministry of Health of the Russian Federation. During the study, 135 samples were sent to the laboratory for genotyping.

### 4.2. PCR and Sequencing

Fecal samples were diluted 1:10 in phosphate-buffered saline (PBS); suspensions were centrifuged at 15,000× *g* for 10 min. An RNA Extraction Kit (Vetbiochem, Moscow, Russia) was used for RNA extraction from the supernatant in accordance with the manufacturer’s instructions. The isolated RNA was used as a template in reverse transcription polymerase chain reactions (RT-PCR) with primers for the VP7 and VP4 genes of rotavirus [38]. A one-tube real-time RT-PCR kit (Alpha Ferment, Russia) was used to perform RT-PCR, in accordance with the manufacturer’s instructions. The amplification products, 861bp for VP7 and 876 bp for VP4, were detected in the 1% agarose gel; the resulting products were gel purified by using a Monarch DNA Gel Extraction Kit (New England Biolabs, Ipswich, MA, USA), following the manufacturer’s instruction. The Sanger reaction was performed by using a Big Dye^®^ Terminator v.3.1 Cycle Sequencing Kit (Applied Biosystems, Waltham, MA, USA), according to the manufacturer instructions, with the same primers. The nucleotide sequences of the genome fragments were assessed with an AB3130 genomic automated analyzer (Applied Biosystems, USA).

### 4.3. Genome Alignment and Phylogenetic Analysis.

The obtained nucleotide sequences were analyzed by using Lasergene 11.1.0. (DNASTAR, Madison, WI, USA). Multiple alignments were performed by using MUSCLE. Phylogenetic dendrograms were inferred by using the maximum likelihood method, GTR model (MEGA 7.0.18). The topology of the trees was confirmed by 1000 bootstrap replication steps [39]. The sequences were compared with those available in the GenBank database by using the Standard Nucleotide BLAST software package (http://www.ncbi.nlm.nih.gov/BLAST, accessed on 16 November 2020).

## 5. Conclusions

The paper presents a study of the diversity of genotypes of rotaviruses circulating in Moscow in the 2019–2020 epidemic season. The most common genotype, similar to the previous season, was G9P[8]. However, for the first time, the G3P[8] genotype took second place. Moreover, all detected viruses of this genotype belonged to Equine-like G3P[8] viruses that had never been detected in Russia before. The presence of rotaviruses with the G8P[8] and G9P[4] genotypes in Moscow was detected for the first time. Among the detected rotaviruses, there were equal proportions of Wa and DS-1 like strains, although previously Wa-like strains accounted for most of the cases studied in Russia. Further monitoring of circulating RVA strains is necessary due to the increasing pressure of vaccines on the evolution of rotaviruses and the high mobility of the population and the associated risk of the introduction of new genotypes.

## Figures and Tables

**Table 1:** Distribution of G/[P]-genotypes in various age groups.

G-Type	[P]-Type	<1 Year	1–2 Years	3–5 Years	6–18 Years	>18 Years	Total
G1	P[8]	1	3	2			6
G2	P[4]		9		1		10
	P[X]		1			1	2
G3	P[8]	1	17	4			22
G4	P[8]		2				2
	P[6]		1				1
G8	P[8]	2					2
G9	P[8]	5	16	8	1	1	31
	P[4]		1		1	1	3
G12	P[8]			1			1
Total		9	50	15	3	3	80

## Supplementary Materials

The following are available online at https://www.mdpi.com/article/10.3390/pathogens10060674/s1, Table S1: GenBank accession numbers assigned for rotavirus genotypes sequenced in this study.
