# Supplementary material for: Prevalence and Genetic Diversity of Group A Rotavirus Genotypes in Moscow (2019–2020)
Source: Pathogens. 2021 May 30;10(6):674. doi: 10.3390/pathogens10060674 (PMC8228337; doi:10.3390/pathogens10060674)
Supplement: Supplementary file 1 [file pathogens-10-00674-s001.zip › pathogens-1181272-supplementary.pdf]

Table 1 Supplementary. GenBank accession numbers assigned for rotavirus genotypes sequenced in this study

Isolate name	G/[P]-genotype	VP7	VP4
Mos-311019-557	G3P8	MT939912	MT939992
Mos-311019-555	G9P8	MT939913	MT939993
Mos-301019-550	G9P8	MT939914	MT939994
Mos-300519-356	G9P8	MT939915	MT939995
Mos-300120-758	G9P8	MT939916	MT939996
Mos-290519-355	G1P8	MT939917	MT939997
Mos-290120-757	G3P8	MT939918	MT939998
Mos-281119-629	G3P8	MT939919	MT939999
Mos-281119-626	G3P8	MT939920	MT940000
Mos-261219-732	G3P8	MT939921	MT940001
Mos-260919-485	G3P8	MT939922	MT940002
Mos-260919-481	G9P8	MT939923	MT940003
Mos-260220-774	G3P8	MT939924	MT940004
Mos-260220-773	G9P8	MT939925	MT940005
Mos-230120-756	G3P8	MT939926	MT940006
Mos-230120-755	G1P8	MT939927	MT940007
Mos-220120-754	G1P8	MT939928	MT940008
Mos-220120-753	G2P4	MT939929	MT940009
Mos-220120-750	G12P8	MT939930	MT940010
Mos-220120-748	G9P4	MT939931	MT940011
Mos-211119-614	G9P8	MT939932	MT940012
Mos-211119-613	G9P8	MT939933	MT940013
Mos-201119-604	G9P8	MT939934	MT940014
Mos-201119-598	G9P8	MT939935	MT940015
Mos-200220-771	G9P8	MT939936	MT940016
Mos-190919-463	G2P4	MT939937	MT940017
Mos-190619-369	G2P4	MT939938	MT940018
Mos-190320-783	G2Px	MT939939	-
Mos-190220-768	G9P8	MT939940	MT940019
Mos-190220-767	G3P8	MT939941	MT940020

Mos-190220-766	G9P4	MT939942	MT940021
Mos-180320-782	G3P8	MT939943	MT940022
Mos-180320-781	G9P8	MT939944	MT940023
Mos-180320-779	G9P8	MT939945	MT940024
Mos-170719-421	G4P8	MT939946	MT940025
Mos-161019-532	G9P8	MT939947	MT940026
Mos-160120-747	G3P8	MT939948	MT940027
Mos-160120-745	G2P4	MT939949	MT940028
Mos-160120-744	G9P8	MT939950	MT940029
Mos-160120-743	G9P8	MT939951	MT940030
Mos-150120-742	G1P8	MT939952	MT940031
Mos-150120-741	G2P4	MT939953	MT940032
Mos-141119-595	G3P8	MT939954	MT940033
Mos-131119-585	G9P8	MT939955	MT940034
Mos-131119-582	G9P8	MT939956	MT940035
Mos-131119-579	G9P8	MT939957	MT940036
Mos-131119-577	G9P8	MT939958	MT940037
Mos-130220-764	G9P4	MT939959	MT940038
Mos-130220-763	G3P8	MT939960	MT940039
Mos-120919-441	G8P8	MT939961	MT940040
Mos-120919-440	G1P8	MT939962	MT940041
Mos-120320-778	G3P8	MT939963	MT940042
Mos-120320-777	G2P4	MT939964	MT940043
Mos-120220-762	G1P8	MT939965	MT940044
Mos-110919-437	G9P8	MT939966	MT940045
Mos-110919-435	G4P6	MT939967	MT940046
Mos-110719-410	G8P8	MT939968	MT940047
Mos-101019-526	G9P8	MT939969	MT940048
Mos-101019-525	G3P8	MT939970	MT940049
Mos-101019-524	G9P8	MT939971	MT940050
Mos-100719-406	G9P8	MT939972	MT940051
Mos-100719-404	G2P4	MT939973	MT940052
Mos-100719-403	G3P8	MT939974	MT940053

Mos-100719-402	G4P8	MT939975	MT940054
Mos-091019-519	G2P4	MT939976	MT940055
Mos-091019-518	G2P4	MT939977	MT940056
Mos-071119-573	G9P8	MT939978	MT940057
Mos-071119-570	G3P8	MT939979	MT940058
Mos-061119-563	G3P8	MT939980	MT940059
Mos-061119-561	G2Px	MT939981	-
Mos-051219-649	G3P8	MT939982	MT940060
Mos-051219-647	G9P8	MT939983	MT940061
Mos-051219-646	G2P4	MT939984	MT940062
Mos-050919-424	G3P8	MT939985	MT940063
Mos-050320-775	G9P8	MT939986	MT940064
Mos-050220-760	G9P8	MT939987	MT940065
Mos-050220-759	G3P8	MT939988	MT940066
Mos-041219-641	G9P8	MT939989	MT940067
Mos-040719-393	G9P8	MT939990	MT940068
Mos-021019-496	G3P8	MT939991	MT940069
